# A survey of mechanical antennas applied for low-frequency transmitting

**DOI:** 10.1016/j.isci.2022.105832

**Published:** 2022-12-20

**Authors:** Yong Cui, Chen Wang, Xiao Song, Ming Wu, Qianyun Zhang, Haiwen Yuan, Zhihong Yuan

**Affiliations:** 1 School of Automation Science and Electrical Engineering, Beihang University, Beijing 100191, China; 2 School of Cyber Science and Technology, Beihang University, Beijing 100191, China; 3 Aerospace CH UAV Co Ltd, Beijing 100074, China

**Keywords:** Telecommunication engineering, Electromechanical System, Mechanical systems

## Abstract

The mechanical antenna (MA) is a new type of low-frequency (LF) transmitting antenna that generates an alternating electromagnetic (EM) signal through the mechanical movement of electric charges or magnetic dipoles, which is an interdisciplinary field including not only antennas but also electromagnetics, materials science, and dynamics. This principle of signaling makes it possible to break the constraints on physical dimensions decided by the wavelength of the traditional antenna so as to achieve LF communications with a smaller size and to provide a novel solution for long-range, underwater, and underground communications, navigation over the horizon, and geological exploring. Therefore, MA has become a research hotspot in the field of LF communications in recent 5 years, and this work proposed a survey on this topic of MA applied for LF transmitting. Firstly, we briefly review traditional low-frequency transmitting antennas and summarize the defect; then we introduce research progress of different implementation schemes for MA, comparing the signaling performance, advantages, and disadvantages of each scheme. Furthermore, we discuss the experiment setup, results, and related technology for MA including signal modulation methods. Finally, we explore prospects for future research about MA. This work presents a comprehensive and critical survey of small LF transmitters based on MA to help the readers to understand and identify the background, status, and challenges of research in this field.

## Graphical abstract

**Figure:**
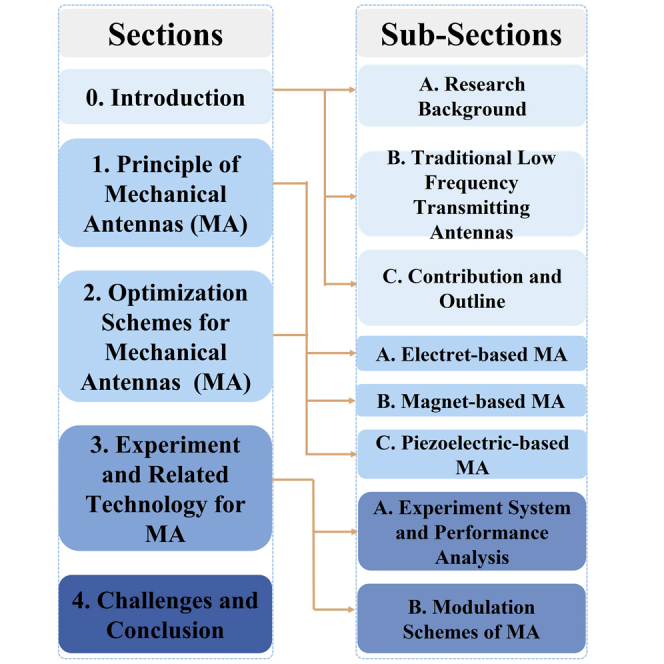

## Introduction

Low-frequency (LF, below 300 kHz) electromagnetic (EM) waves have the characteristics of strong stability, low attenuation in the medium, and long propagation distance (Al-Shamma’a et al., 2004; Tahir et al., 2018). They have been widely used in geological survey, seismic research, navigation, underground communication, and submarine communication. Especially in the field of submarine communication, LF electromagnetic waves play an indispensable role.

Effective submarine communication, especially with nuclear submarines, is a crucial link for ensuring sea-based strategic deterrence and tactical strikes. However, since seawater is a good conductor, its skin effect is obvious, and the attenuation of electromagnetic waves in seawater is very significant (Wang et al., 2014a, 2015). The higher the frequency, the faster the attenuation. Therefore, the high-frequency (HF, above 3 MHz) electromagnetic wave communication system widely used on land cannot be realized underwater, and a submarine needs to drag a communication buoy to the water’s surface to receive signals. However, wartime application scenarios for submarine communications determine the hidden and survivable requirements of this technology. With communication buoys, it is relatively easy for the enemy to obtain the position of the submarine, and thus it is not suitable for use in wartime.

Meanwhile, acoustic wave communication has weak anti-interference ability and is mainly used in underwater-to-underwater communications (Erol-Kantarci et al., 2011; Huang et al., 2018; Qiao et al., 2017; Stojanovic and Preisig,2009; Xia et al., 2014). Blue-green laser communication is often unidirectional and still in the research stage (Chen et al., 2017; Kaushal and Kaddoum,2016; Oubei et al., 2015; Wu et al., 2017; Zeng et al., 2017). Therefore, LF electromagnetic waves are more desirable for submarine communication.

Several countries have established LF transmitting stations. Germany built the “Goliath” very-low-frequency (VLF, 3–30 kHz) transmitting system in Saxony. The United States built a VLF submarine communication network capable of global communication to the mainland, Hawaii, Puerto Rico, Japan, the United Kingdom, Iceland, Turkey, and Australia, etc.

The Wisconsin Transmitter Facility (WTF)/Michigan Transmitter Facility (MTF) super-low-frequency (SLF, 30–300 Hz) transmitting system was also developed by the United States (Lu, 2013). Meanwhile, Russia built “ZEVS” SLF transmitting system in Kola Peninsula. Certain parameters of traditional LF transmitting antenna above are shown in Table 1.

**Table 1:** Certain parameters of traditional LF transmitting antenna

Name	Country	Frequency Range	Size	Power Consumption
Goliath	German	15–25 kHz	2.4 km	1.8 MW
WTF/MTF	U. S. A	SLF	>260 m	1.8 MW
ZEVS	Russia	SLF	10.5 km	>10 MW
HAARP	U. S. A	ELF to VLF	23.5 ha	3.6 MW

**Figure 1:**
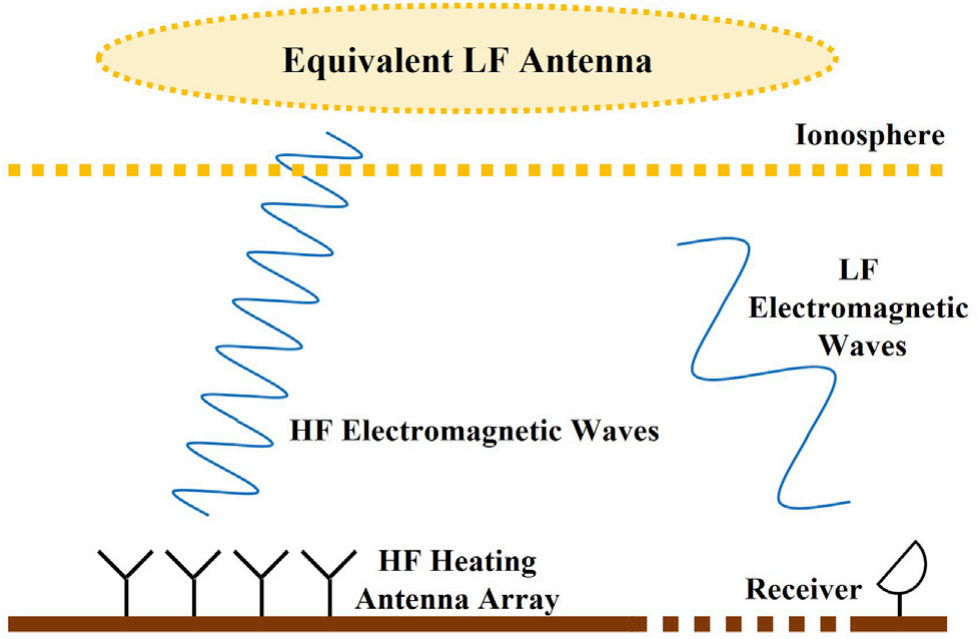
HF-modulated heating of ionosphere to generate LF radiation

The use of high-power HF electromagnetic waves to artificially heat the ionosphere and generate LF radiation is another implementation of LF transmitting systems as shown in Figure 1, which can produce electromagnetic waves ranging from extremely low frequency (ELF, 3–30 Hz) to VLF ranges (Chang et al., 2011, 2014; Maxworth et al., 2015; Moore et al., 2012, 2013). Major countries around the world have also established high-power HF ionosphere-heating devices such as HIPAS, Arecibo, Tromsφ, and HAARP (Cohen and Golkowski, 2013).

However, there are several problems existing in the traditional LF transmitting system. Owing to the long wavelength of LF signal, to achieve effective electromagnetic radiation, the land-based LF transmitting systems besides the high-power HF heating ionosphere method must have big volume of the kilometer (km) scale and large energy consumption of the megawatt (MW) scale, which can be seen from Table 1. What’s more, for the factors of the grounding network, the LF transmitting systems have extremely high on-site requirements, which are mostly built in areas with low earth conductivity and have high requirements in terms of geological structure and geographical environment. An LF transmitting system, despite its large scale, remains an electrically small antenna (Best, 2015; Lopez,2008; McLean, 1996), so it has a low radiation efficiency (Thiele et al., 2003; Underhill and Harper, 2003). Meanwhile, as survivable and mobile schemes, the airborne dual trailing antennas and the vehicle balloon lifting antennas still have large and obvious external targets, which results in poor viability and are expensive to maintain (Wang et al., 2014b). These defects limit the application of LF communication.

In order to solve the problems of the existing LF transmitting systems, the defense advanced research projects agency (DARPA) launched the A MEchanically Based Antenna (AMEBA) project in 2017 to study small, lightweight, and low-power solutions for LF transmitting antennas (Ding, 2017). The term mechanical antenna (MA) arises from the AMEBA technology, which is different from the general definition of MA that a dish antenna or other whip like antenna can be mechanically configured. MA can be carried on airplanes, unmanned aerial vehicles, airships, satellites, vehicles, land stations, ships, and submarines or even carried on the body, building an integrated LF military communication network with the sky, the earth, and the sea. They can be arranged anywhere in the city to achieve precise indoor positioning and have great civilian value. In short, the transmitting antennas based on MA have bright development and application prospects.

At present, a lot of institutions are devoted to the field of MA and have carried out various research works on the theoretical models, implementation schemes, optimized designs, and signal-modulation schemes of MA.

**Figure 2:**
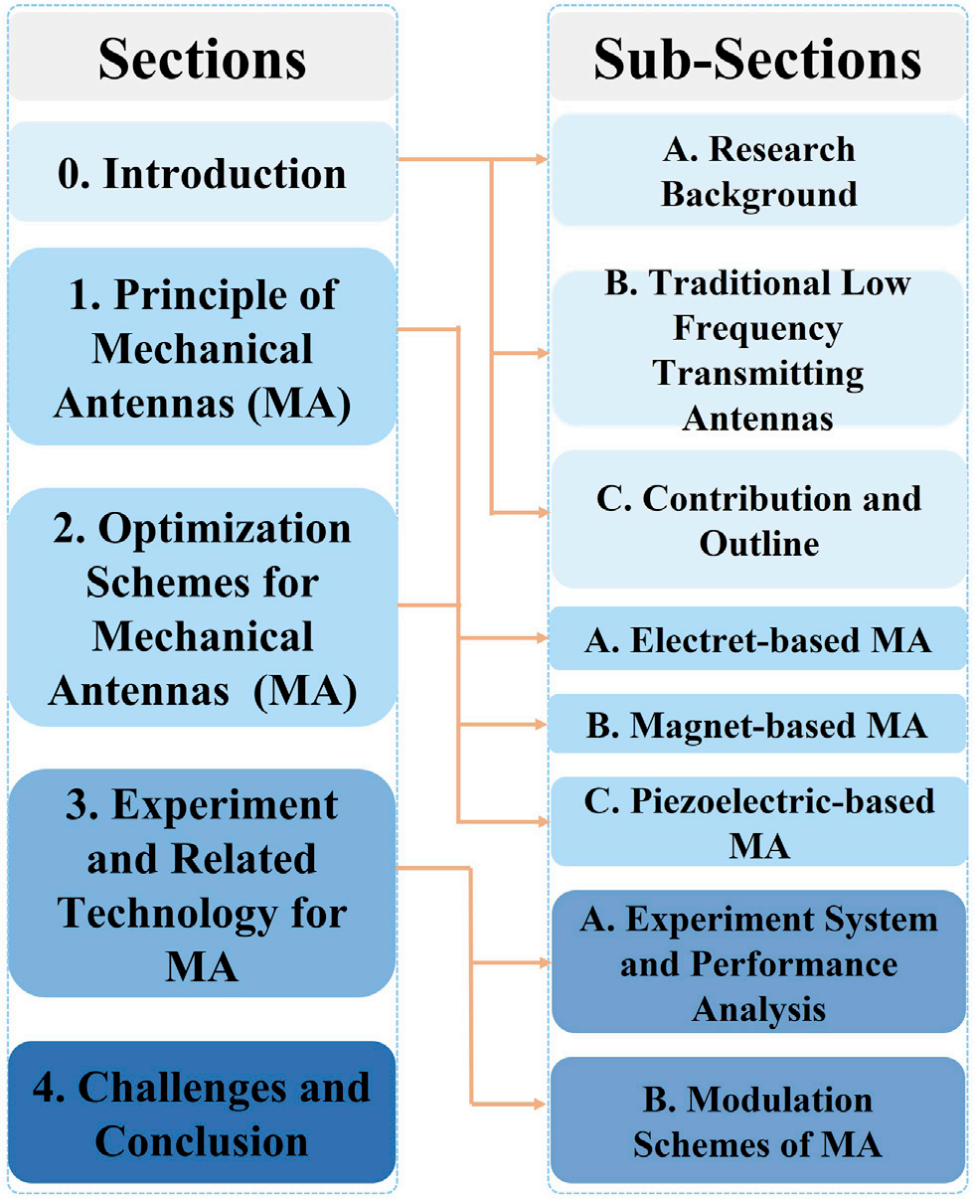
Structure of small LF transmitting antennas survey

To present readers a comprehensive understanding of the research of this research filed, this work surveys the literature about the LF transmitter based on MA in nearly 5 years (2017–2022) from the launching of the AMEBA project and briefly introduces the traditional LF transmitting antennas and electromagnetic wave receiving devices. The structure of this work is shown in Figure 2.

In section principle of mechanical antennas and optimization schemes for mechanical antennas, we divide MA into electret-based, magnet-based, and piezoelectric-based and discuss the development of MA. We summarize the current research progress in this field, describe the principles and implementation of existing MA, and sort out the innovations of various schemes.

In section experiment and related technology for MA, we provide the experiment setup, results, and related technology for MA including signal modulation methods and common LF electromagnetic wave receiving methods. We compare the performance of existing MA solutions and discuss the development direction of miniaturization of receiving devices.

In section conclusions and future research directions, we provide the conclusion of the survey and discuss the research focus in the field of small LF transmitting antennas in the next stage.

## Principle of mechanical antennas

Unlike traditional antennas, MA does not rely on the oscillating current of electronic circuits to generate radiation but uses mechanical energy to drive the movement of charges or magnetic dipoles. A permanent magnet has a magnetostatic field around it, and so if the magnet undergoes oscillatory motion, a fixed point in the vicinity of the magnet will experience an oscillating magnetic field. In the alternative scheme, when a small electric charge experiences motion, it produces circles of magnetic field around its axis of motion according to the Biot-Savart Law. If the small charge is experiencing oscillatory motion, then the magnetic field at a fixed point in the vicinity of this charge will also experience an oscillating magnetic field. These two signaling schemes can be exploited to enable near-field signaling at frequencies which are too low to be utilized by traditional antennas and have the extra advantage that one does not need a huge impedance-matching network, and so one can miniaturize LF communication equipment (Prasad et al., 2017; Nagaraja and Prasad, 2017).

At present, from the key MA materials, existing MA research programs can be divided into three types: electret-based MA (Wang et al., 2021a, 2021b; Zhou et al., 2021), magnet-based MA (Guo et al., 2021; Han et al., 2021; Liu et al., 2021; Wang et al., 2021c), and piezoelectric-based MA (Cao et al., 2022; Xu et al., 2021; Zaeimbashi et al., 2021).

### Electret-based MA

Electret is a dielectric material that can store space charge or electric dipole. Its polarization characteristics do not disappear completely with the removal of the applied electric field. It has a long relaxation time and remains in a metastable polarization state for a long time (Erhard et al., 2010). Common electret materials include inorganic electret materials such as SiO_2_ and organic electret materials such as polytetrafluoroethylene (PTFE) and fluorinated ethylene propylene (FEP) (Xia et al., 2005). Through different polarization modes, a single charge or dipole can be stored in the electret. When the polarized electret material moves in a simple harmonic motion, the bound charges inside it also move, thereby generating external radiation (Ding and Song, 2019).

The motion modes of electret-based MA include vibration and rotation. The vibration-based electret MA generates vibration through a driver and transmits the vibration to the electret. The vibration of the electret drives the vibration of its internal charge, thereby generating radiation of the corresponding frequency. At present, of the vibration-based electret MA schemes, Youjun Tian form Honeywell used a micro-electro-mechanical system (MEMS) electric driver as the vibration source (Tian, 2019), while Huan Zheng et al. from the China State Shipbuilding Corporation Limited (CSIC) 722nd Research Institute used piezoelectric ceramics as the vibration source, combining an electret-based MA with a piezoelectric-based MA and generating vibration through the piezoelectric effect of the piezoelectric ceramics (Zheng et al., 2018).

As to rotation-based electret MA, Zongxin Wang et al. from Southeast University, China, has modeled and simulated them based on the rotating parallel plate capacitor (Wang et al., 2018). Yong Cui et al. from Beihang University and Minsong Wei from the University of California, Berkeley, also conducted a lot of research (Cui et al., 2019a).

James A. Bickford et al. from Draper conducted a simulation study on antenna performance under the two motion modes of vibration and rotation and concluded that the rotation-based electret MA has better radiation efficiency (Bickford, 2019; Bickford et al.,2017, 2019).

As for the model of a flat, rotation-based electret MA, the electret materials with single positive or negative charges are symmetrically distributed on the disk (Cui et al., 2018a). When the electret rotates with the disc, the current elements formed by the opposite charges on any two symmetric electrets around the center of the circle have the same direction, and the generated magnetic fields are superimposed on each other.

Integrating the magnetic field generated by each current element of the rotating electret at the receiving point can provide the magnetic flux density *B* of the flat rotation-based electret MA (Cui et al., 2019b):

(Equation 1) $$B=2\iint dB=2\cdot \frac{\mu_{0}\rho \omega }{4\pi r^{2}}\cdot \int_{0}^{R}R^{2}dR\int_{-\frac{\pi }{2}}^{\frac{\pi }{2}}\;\cos\;\theta d\theta =\frac{\mu_{0}\rho \omega R^{3}}{3\pi r^{2}}$$

where $\mu_{0}$ is the permeability of the vacuum, ρ is the charge density of the electret, ω is the rotational angular velocity of the antenna, *R* is the radius of the antenna, and *r* is the distance from the receiving point to the antenna. It can be seen that the magnetic flux density of the rotation-based electret MA is inversely proportional to the square of the distance and proportional to the rotational angular velocity of the antenna and the electret charge density.

**Figure 3:**
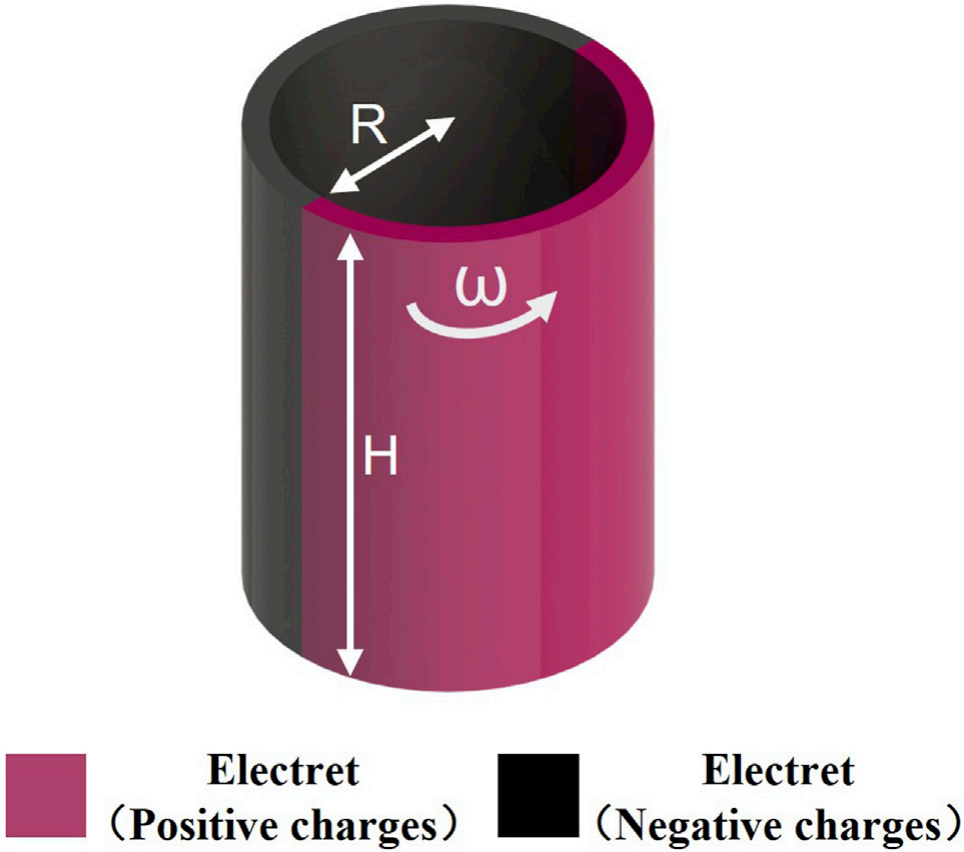
Model of cylindrical rotation-based electret MA

Integrating the magnetic field generated by the flat rotation-based electret MA into *z* axis can provide the magnetic field propagation model of the three-dimensional cylindrical rotation-based electret MA, as shown in Figure 3 (Wang et al., 2020a). Ignoring the size of the transmitting antennas, for any signal receiving point P(x,y,z) in the space,

(Equation 2) $$B=\left|\frac{\mu_{0}\rho HR}{2\pi r^{2}}\int_{-\frac{\pi }{2}+\omega t}^{\frac{\pi }{2}-\omega t}\vec{v}\times \vec{l}d\theta \right|$$

(Equation 3) $$\vec{v}\times \vec{l}=\frac{\omega R}{r}\left(z\;\cos\;\theta ,-z\;\cos\;\theta ,y\;\sin\;\theta -x\;\cos\;\theta \right)$$

So, the relationship between the magnetic flux density and the distance, speed, and charge density of the cylindrical rotation-based electret MA is the same as that of a flat rotation-based MA.

### Magnet-based MA

A common magnet-based MA scheme is to drive the NdFeB magnet to rotate using a motor to generate magnetic field radiation corresponding to the rotation frequency (Gong et al., 2018; Madanayake et al., 2017; Yi et al., 2020; Ren et al., 2020). The magnet can be regarded as a collection of countless magnetic dipoles. The analysis of the rotating-magnet-based MA mostly starts from the theory of rotating magnetic dipoles.

Qiang Zhou and Wei Shi from the 63rd Research Institute of the National University of Defense Technology carried out systematic modeling analysis and theoretical research on the radiation principles of various MA (Shi et al., 2019; Zhou et al.,2020a, 2020b). The rotating magnetic dipole antenna is equivalent to a current loop model. The radiation model in spherical coordinates is as follows:

(Equation 4) $$B=\frac{\mu_{0}k^{3}m_{0}}{4\pi }e^{-jkr}\left\{2\left[\frac{j}{{\left(kr\right)}^{2}}+\frac{1}{{\left(kr\right)}^{3}}\right]\cos\;\theta \hat{r}-\left[\frac{1}{kr}-\frac{j}{{\left(kr\right)}^{2}}-\frac{1}{{\left(kr\right)}^{3}}\right]\sin\;\theta \hat{\theta }\right\}$$

where *k* is wavenumber and $m_{0}$ is magnetic dipole moment.

When the antenna works in a near-field area, ≪1 , the radiation model of the rotating-magnet-based MA can be approximated as

(Equation 5) $$B=\frac{\mu_{0}m_{0}}{4\pi r^{3}}\cdot \left[2\left(\cos\;\theta +j\;\sin\;\theta \;\sin\;\varphi \right)\hat{r}+\left(\sin\;\theta -j\;\cos\;\theta \;\sin\;\varphi \right)\hat{\theta }-j\;\cos\;\varphi \hat{\varphi }\right]$$

It can be seen that the magnetic flux density of the rotating-magnet-based MA in the near-field area is inversely proportional to the third power of the distance. So the field variation with distance is the same as it would be if the magnet were stationary.

### Piezoelectric-based MA

Unlike the above two rotating MA, the piezoelectric-based MA uses vibration generated by piezoelectric materials as the main source of magnetic field radiation.

At present, there are two approaches to achieve piezoelectric-based MA. One is a piezoelectric MA that uses both the positive and inverse piezoelectric effects of piezoelectric materials to generate radiation, and the other is a magnetoelectric (ME) MA that uses the magnetoelectric effect possessed by the magnetoelectric heterostructure, combining piezoelectric material and magnetostrictive material to generate radiation (Chen et al., 2020).

**Figure 4:**
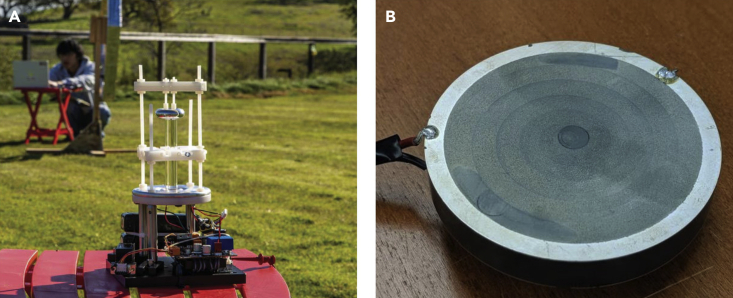
Piezoelectric mechanical antennas (A and B) designed by Mark A. Kemp et al. from SLAC National Accelerator Laboratory; (B) designed by Ahmed E. Hassanien et al. from University of Illinois at Urbana-Champaign. Figures reproduced from (A) (Kemp et al., 2019) Copyright 2019, Nature Publishing Group; (B) (Hassanien et al., 2020a) Copyright 2020, Nature Publishing Group.

Piezoelectric MA uses piezoelectric crystals, piezoelectric ceramics, and other piezoelectric materials as the main core unit (Kemp et al., 2018). The piezoelectric material is excited by an external alternating electric field, so it produces a deformation that changes with external excitation and drives the movement of the bound charge on the surface of the piezoelectric material and the internal dipole moment to form a dipole current, thereby generating external radiation. Two kinds of piezoelectric MA are shown in Figure 4.

**Figure 5:**
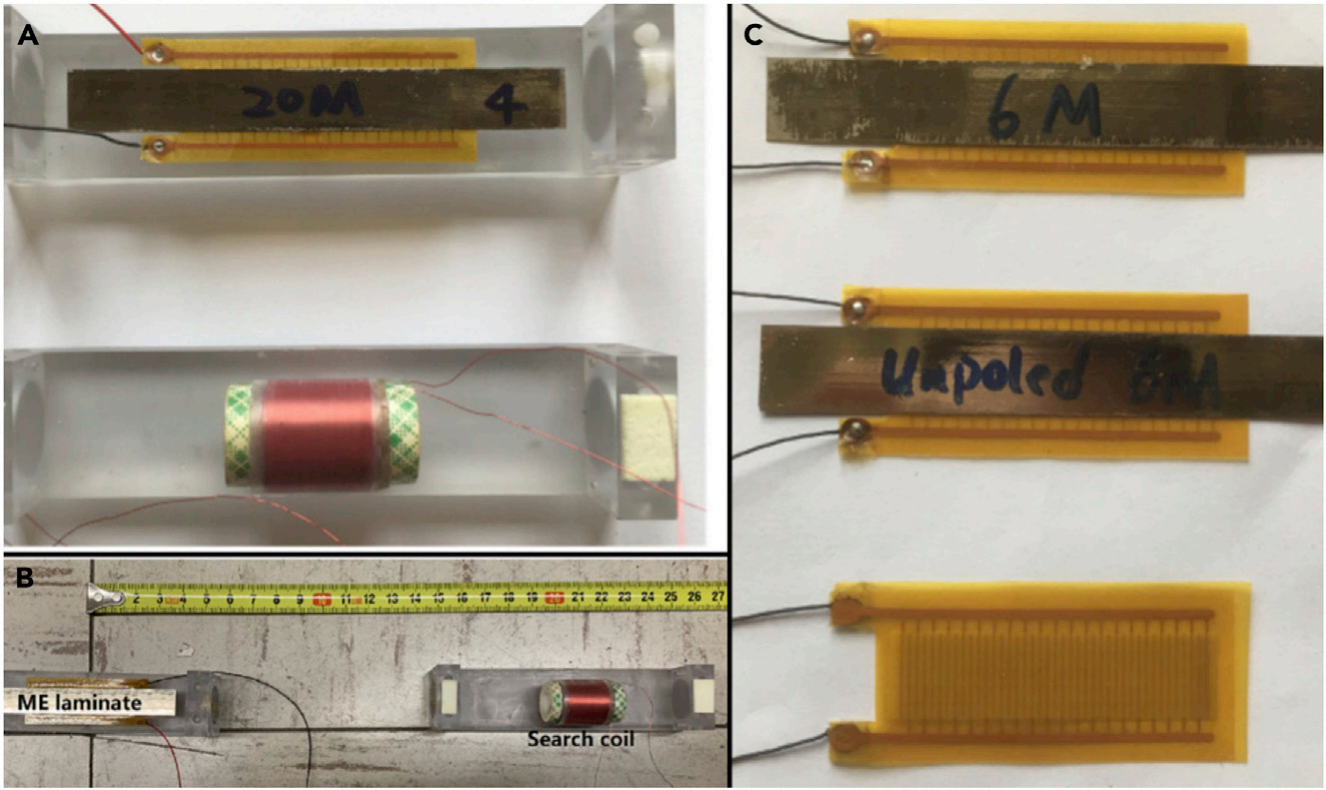
Structural of magnetoelectric MA (A–C) fabricated ME transmitter and search coil used for test and measurements; (B) three samples- 6 Metglas layers (6M)-based ME transmitter, unpoled 6M ME transmitter and poled Piezo layers used in characterization; (C) experimental set-up for field measurements. Figures reproduced from: (A–C) (Xu et al., 2019a) Copyright 2019, Multidisciplinary Digital Publishing Institute (MDPI).

Magnetoelectric MA incorporates a magnetic and piezoelectric heterostructure, which can combine the piezoelectric effect with the magnetostrictive effect (Schneider et al., 2019). The magnetic film can sense H-components of EM waves and then induce an oscillating strain, which will generate a piezoelectric voltage output at the electromechanical resonance frequency (Zaeimbashi et al.,2021). This principle was used in the very-high-frequency (VHF, 30–300 MHz) and ultra-high-frequency (UHF, 0.3–3 GHz) bands, using electromechanical resonance instead of electromagnetic resonance to break the size and efficiency limitations of traditional small antennas (Nan et al., 2017). This concept can be applied in LF bands as well. The structure of the magnetoelectric MA researched by Junran Xu et al. from Virginia Tech is shown in Figure 5: a sandwich structure of magnetostrictive layer-piezoelectric layer-magnetostrictive layer (Dong et al., 2020; Xu et al., 2019a). The piezoelectric layer in the middle generates a piezoelectric effect under the excitation applied by the electrode and produces vibration of the same frequency as the alternating excitation signal, which drives the upper and lower magnetostrictive layers to vibrate, thereby generating magnetic field radiation. During the vibration transmission process, the vibration frequency does not change, so the frequency at which the antenna radiates magnetic signals to the outside is the vibration frequency of the piezoelectric material. In addition, the magnetoelastic coupling plays a direct role in determining performance of the magnetoelectric MA. Kevin Q. T. L. and Yuanxun Wang from the University of California at Los Angeles approached the analysis of magnetoelastic coupling accurately, quantified the coupling by defining magnetoelastic coupling coefficients, and derived conditions of applied stress to maximize it (Luong and Wang, 2022).

## Optimization schemes for mechanical antennas

The optimization of an MA mainly consists of increasing the frequency range and intensity of the radiation (Table 2). For the former, the rotating MA is limited by the performance of the motor speed and torque, and it is difficult to increase the rotation speed significantly. Bypassing the limitations on motor performance and increasing the frequency of the magnetic field are important components of MA optimization research. The frequency range of piezoelectric-based MA is also limited by the material-processing technology and the characteristics of the piezoelectric materials used. The latter can be optimized according to the characteristics of various types of MA.

**Table 2:** Optimization schemes of mechanical antennas

Optimization purpose	MA type	Optimization method
Increase frequency range	Electret-based MA	Multi-block electret-based MA structure
	Magnet-based MA	Orthogonal bow-tie structure
		Magnetic pendulum array MA
		Static permanent magnet, rotating soft magnetic material
		Static permanent magnet, coil current modulates reluctance
	Piezoelectric-based MA	Use the customized macro fiber composite (MFC) as the magnetoelectric material
Increase radiation intensity	Electret-based MA	Use mechanical amplifier to increase vibration amplitude
		Increase the antenna volume by increasing the radius
	Magnet-based MA	Use external magnetic poles to increase magnetic dipole moment
	Piezoelectric-based MA	Work in a vacuum chamber
		Choose piezoelectric materials with high dielectric constant

### Electret-based MA

**Figure 6:**
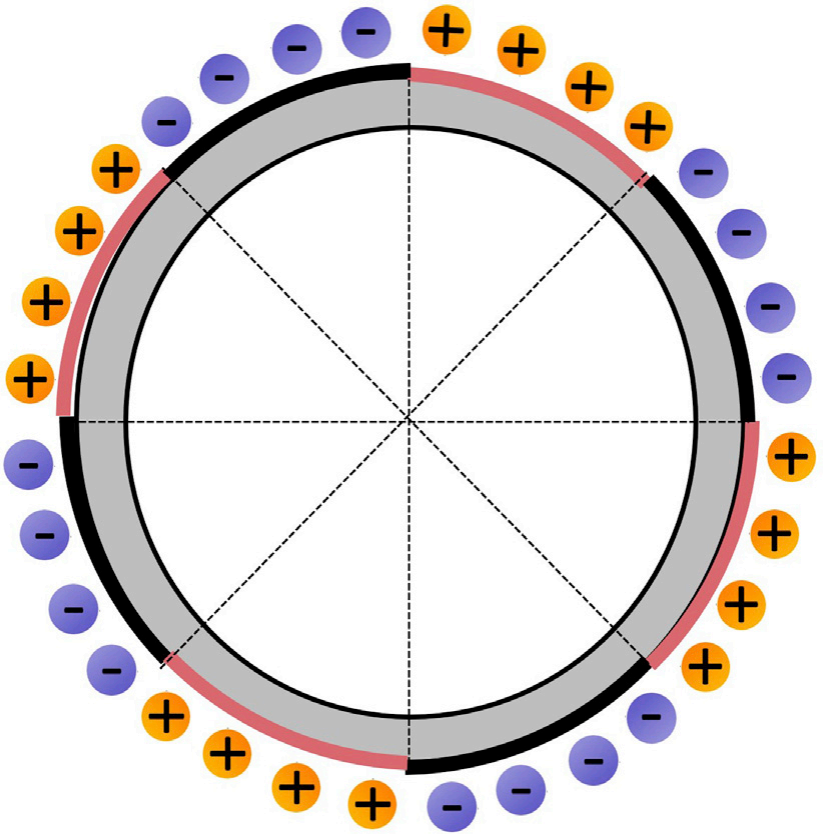
The structure of multi-block electret-based MA（8 distribution）

In order to increase the radiation frequency of the electret-based MA, Bowen Liang et al. from Beihang University designed a multi-block electret-based MA (Liang et al., 2019). When changing the distribution of the electret film on the rotating cylinder, as shown in Figure 6, the radiation produced by the antenna also changes. The simulation analysis shows that as the number of electret films increases, so does the frequency of the magnetic field; however, the maximum magnetic flux density at the same signal receiving point continues to decrease, and the maximum magnetic flux density attenuates faster.

In order to increase the radiation intensity, Youjun Tian and Huan Zheng et al. added mechanical amplifiers such as a girder and rod to their vibrating-electret-based MA, which amplifies the vibration generated by the vibration source and transmits it to the electret, thereby increasing the vibration amplitude.

For electret-based MA, the amount of charge determines the radiation intensity. Under the premise that charge density is difficult to increase, instead increasing the volume is an effective way to increase the radiation intensity. For a cylindrical rotating-electret-based MA, the shape parameters such as height and radius will affect the performance of the antenna. Theoretical research and simulation analysis show that the magnetic flux density increases linearly with an increase in height and increases quadratically with an increase in radius. Therefore, when the area of the electret attached to the antenna surface is the same, increasing the radius of the antenna is more beneficial to increasing the radiation intensity. At the same time, the mass distribution of the short and thick antenna makes its mechanical structure more stable, which facilitates the work of the rotating antenna.

### Magnet-based MA

**Figure 7:**
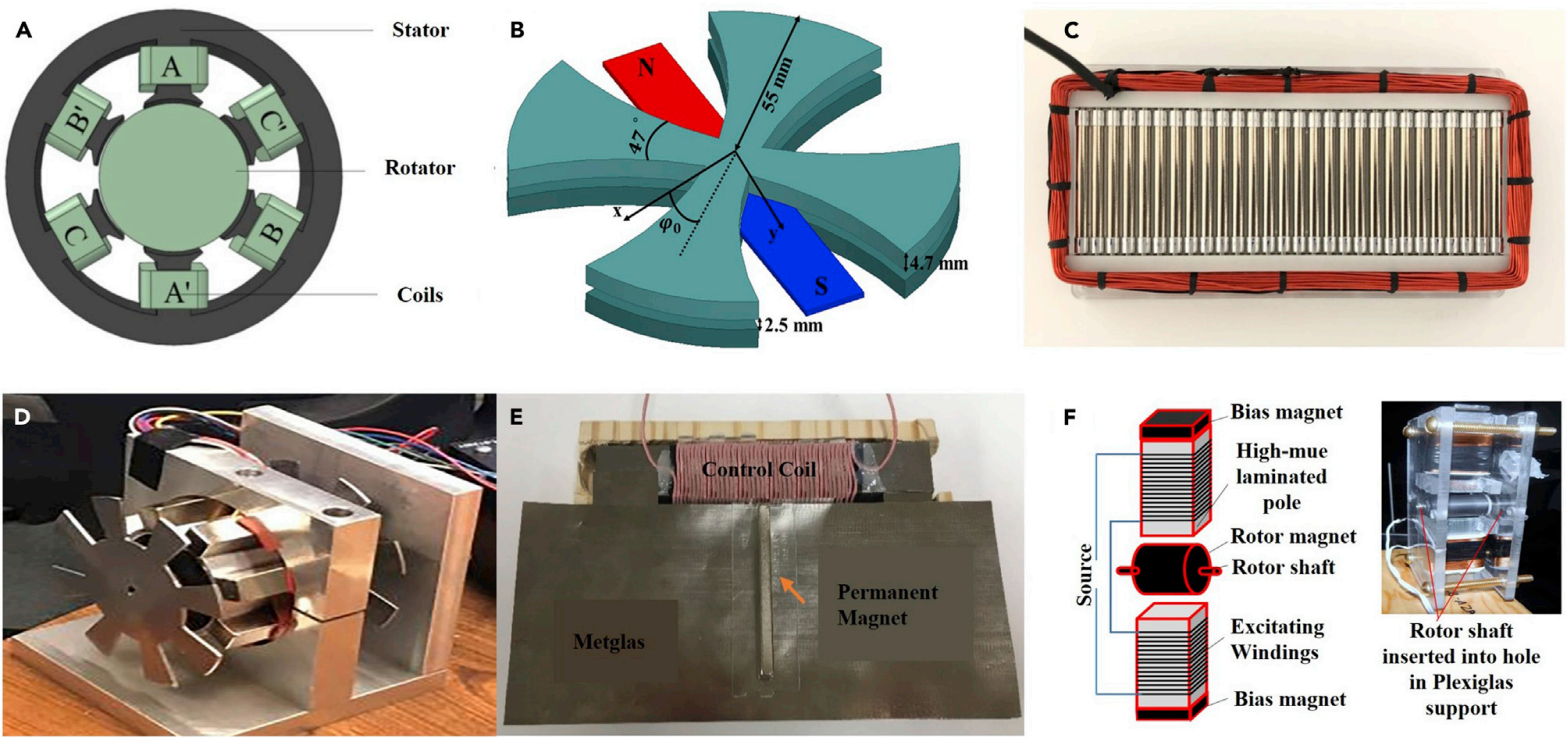
Typical optimization schemes for a magnet-based MA (A–F) based on three-phase induction motor, designed by Yunxiao Xu from Shanghai Jiao Tong University; (B) increase the radiation frequency with a bowknot structure, designed by Navid Barani and Kamal Sarabandi from University of Michigan; (C) magnetic pendulum array, designed by Srinivas Prasad M N  et al. from University of California, Los Angeles; (D) distort the magnetic field generated by the permanent magnets, designed by Mark Gołkowski et al. from University of Colorado Denver; (E) modulate the current in the control coil to change the direction Han et al. (2021) bn of the magnetic flux and the storage position of the magnetic energy, designed by Hossseini-Fahraji et al. from Virginia Tech; (F) increase the magnetic dipole moment, designed by Olutosin Charles Fawole et al. from University of Utah. Figures reproduced from: (a) () Copyright 2021, IEEE; (b) (Barani and Sarabandi,2019) Copyright 2019, IEEE; (c) (Prasad et al., 2019a) Copyright 2019, Nature Publishing Group; (d) (Golkowski et al., 2018) Copyright 2018, IEEE; (e) (Hosseini-Fahraji et al., 2021) Copyright 2021, IEEE; (f) (Fawole and Tabib-Azar, 2017) Copyright 2017, IEEE.

For a rotating MA, the motor is the driver of mechanical motion, and the working principle of the motor determines that it also generates magnetic field radiation corresponding to the rotating frequency. This magnetic field radiation is often superimposed on the magnetic field radiation generated by the electret or magnet in the MA. Based on this, as shown in Figure 7A, Yunxiao Xu and Junping Geng from Shanghai Jiao Tong University designed a magnet-based MA with a three-phase induction motor, which generates magnetic field radiation directly through the motor instead of driving the external permanent magnet (Han et al., 2021; Xu, 2019; Xu et al., 2019b).

In order to increase the radiation frequency, similarly to the scheme where a rotating-electret-based MA has the distribution of electret films of different polarities changed to achieve signal frequency doubling, Navid Barani and Kamal Sarabandi from the University of Michigan proposed a special structure for the rotating-magnet-based MA, as shown in Figure 7B. With the expense of less radiated power, It multiplies the frequency of the radiated signal (Barani and Sarabandi, 2019; Barani et al., 2018). In this scheme, the permanent magnet is placed between two pairs of orthogonal bow-tie-shaped high-permeability materials, and the phase modulation is realized by changing the initial relative position of the permanent magnet and the orthogonal bow-tie structure. When the permanent magnet rotates through the gaps of the orthogonal bow-tie, the one-period signal approximately changes to multiple periods, but at the same time, the signal amplitude doubles. Similarly, Faxiao Sun et al. from University of Chinese Academy of Sciences manufactured a prototype of the rotating shutter antenna and has achieved indoor ultra low frequency (ULF) communication with a code rate of 12.5 bps (Sun et al., 2022).

On the basis of the conventional single-rotating-magnet-based MA, Srinivas Prasad M N  et al. from the University of California at Los Angeles proposed an array of rotating-magnet-based MA, as shown in Figure 7C, and finally developed a 2D stacked magnetic pendulum array MA (Prasad et al., 2018a, 2018b, 2019a, 2019b; Selvin et al., 2017; Mysore Nagaraja et al., 2019; Fereidoony et al., 2021). This scheme no longer uses a motor to drive the rotation of the permanent magnets but aligns the magnets through the mutual influence of the magnetic fields of multiple permanent magnets in the pendulum array. Then the movement of the magnetic pendulum array is excited by the alternating current (AC) magnetic field, in turn excited by the external coil. This scheme uses multiple small permanent magnets instead of a single large permanent magnet, which reduces the mechanical energy density and increases the electromagnetic energy density. In addition, using coil excitation to make the magnet swing removes the limitation of the motor in the conventional scheme so that the antenna can have a larger frequency adjustment range; the conversion of mechanical driving to electric driving brings greater convenience to signal modulation. Similarly, Rhinithaa P. Thanalakshme et al. from University of Illinois at Urbana-Champaign explored a resonant magnetomechanical transmitter which can produce and scale detectable ULF magnetic fields with frequencies ranging from sub-100 Hz to above 1 kHz (Thanalakshme et al., 2021).

Unlike other schemes that directly drive the movement of the permanent magnets to generate radiation, Mark Gołkowski et al. from the University of Colorado at Denver chose to rotate soft magnetic materials near several fixed and independent permanent magnets to distort the magnetic field generated by the permanent magnets, as shown in Figure 7D, thereby generating the required magnetic signal (Golkowski et al., 2018). This scheme reduces the contradiction between the high radiation intensity of the conventional rotating magnet schemes and the high load generated by the large-mass permanent magnet on the motor and can easily increase the radiation intensity by increasing the number of permanent magnets. Similarly, Daniel J. Orfeo et al. from the University of Vermont proposed a novel design of Y-Stator vibrating MA, whose principle is that soft-magnet stators can project magnetic fields generated by the vibrating permanent magnets and switch the overall magnetic dipole moment direction in each cycle (Orfeo et al., 2021).

Ali Hossseini-Fahraji et al. from Virginia Tech also chose a static permanent magnet scheme, as shown in Figure 7E (Manteghi, 2017; Hosseini-Fahraji et al., 2021). By modulating the current in the control coil, changing the material instead of the structure, the magnetoresistance in the magnetic field generated by the stationary permanent magnet is modulated, thereby changing the direction of the magnetic flux and the storage position of the magnetic energy and then generating time-varying magnetic field radiation.

In order to further improve the radiation intensity of the rotating-magnet-based MA, Olutosin Charles Fawole et al. from the University of Utah have set two magnetic poles at the radial positions of the rotating magnet to increase the magnetic dipole moment of the permanent magnet (Fawole and Tabib-Azar, 2017). Two magnetic poles are also set in the axial position to realize the adjustment of the magnetic dipole moment, as shown in Figure 7F.

### Piezoelectric-based MA

The radiation intensity of an antenna is determined by the maximum achievable current and its distribution (Hassanien et al., 2020a, 2020b). For the piezoelectric MA, the maximum current is related to the charge density. The greater the charge density, the greater the radiation intensity. When the charge density reaches a certain level, the electric field intensity around the antenna will reach the breakdown limit of air, according to Ahmed E. Hassanien (Hassanien et al., 2020b). The electric field intensity is directly proportional to the charge density and inversely proportional to the relative permittivity of the piezoelectric material. Therefore, for piezoelectric materials with low relative permittivity, the upper limit of the radiation intensity generated is lower. In order to improve the radiation intensity, Mark A. Kemp et al. chose to place the LiNbO_3_ crystal, which is the radiation unit of the antenna, in a vacuum chamber to solve the problem of the breakdown limit of air (Kemp et al., 2019). Ahmed E. Hassanien et al. chose piezoelectric ceramics (PZT) piezoelectric material with a relatively large dielectric constant as the radiation body, thereby increasing the charge density (Hassanien et al., 2020b).

Limited by the material-processing technology, existing piezoelectric materials make it difficult to construct larger piezoelectric-based MA (Pradhan et al., 2020), so the effective radiation area is scant, and it is hard to increase the radiation intensity by increasing the volume. For magnetoelectric MA, the rigidity matching of piezoelectric materials and magnetostrictive materials is also a major difficulty in antenna research.

Using the customized macro fiber composite (MFC) as a magnetoelectric material is a feasible scheme. Using large-strain MFC, through composite structure and interface control, optimizing electric field distribution and interface stress transmission, a magnetoelectric MA of larger size, lower frequency, and larger bandwidth can be realized (Deraemaeker et al., 2009; Kovalovs et al., 2009; Li et al., 2016; Lin et al., 2013; Shahab and Erturk, 2017; Varghese et al., 2015; Zhang et al., 2015, 2017).

The performance of piezoelectric-based MA can also be improved by increasing the number of transmitting unit as well. Yiming Zhang et al. proposed that the transmitter composed of 3 MA units with the series-parallel connection can broaden the operation frequency range, and the intensity of the radiated magnetic field is 2.6 times that of a single unit (Zhang et al., 2022).

## Experiment and related technology for MA

### Experiment system and performance analysis

MA experiments serve mainly to verify whether an antenna can generate magnetic field radiation with a specific frequency and to measure how much magnetic field radiation can be generated at a certain distance.

**Figure 8:**
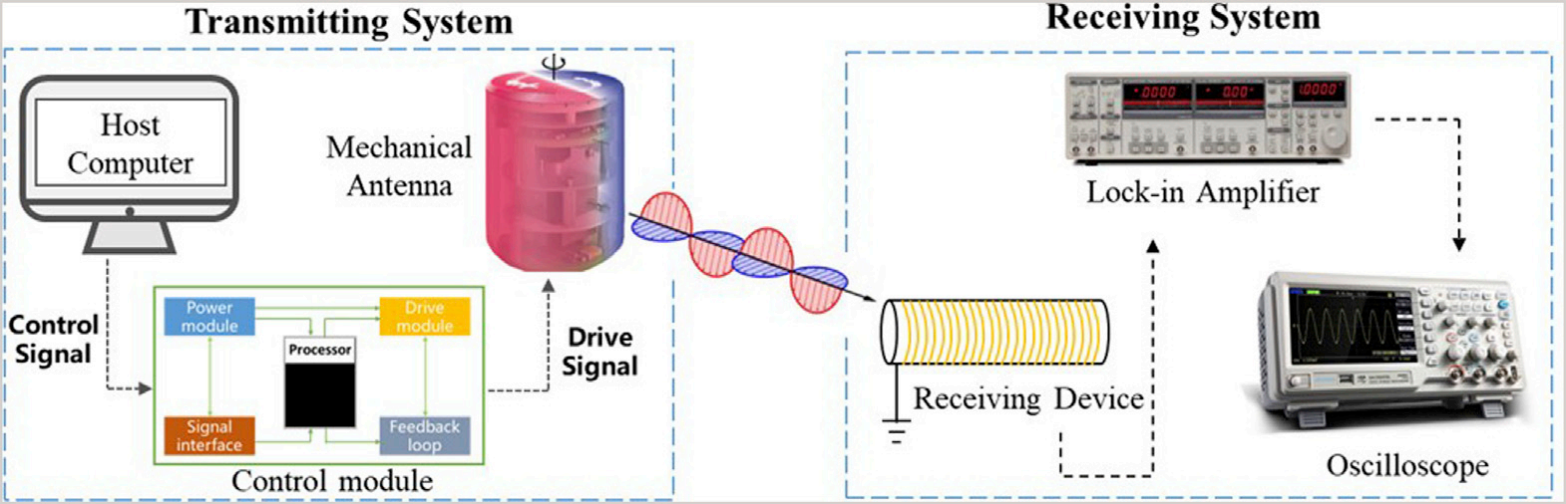
System of transmitting and receiving signals in experiments

The experimental system consists of two parts: transmitting and receiving, as shown in Figure 8.

The transmitting system is composed of units such as the host computer, control module including power supply and drive control, and MA. The receiving part is composed of units such as the receiving device, lock-in amplifier, and oscilloscope. According to the differences in the focus of each program, the specific components of the experimental system are slightly different, but the functions they achieve are basically the same (Burch et al., 2018; Cao et al., 2019; Tarek et al., 2018; Wang et al., 2020b).

The host computer provides a signal source for the MA through the control module so that the motor or piezoelectric motion module in the antenna produce a given frequency of movement, and the MA generates magnetic field radiation.

The receiving device receives the magnetic field signal. Coil and magnetic field sensor are commonly used signal-receiving devices in MA experiments. The lock-in amplifier extracts a specific frequency signal from the mixed signal then outputs it to the data display modules such as the oscilloscope.

Table 3 shows the results of experiments in various references. In general, research teams have achieved LF communication at a range of 1 km with relatively low efficiency compared with HF transmitting antennas, but it is still higher than that of traditional LF transmitting antennas under the same condition [68], which can verify the superiority of the MA convincingly. MA projects are still in the laboratory research stage and have much space for enhancement. The future devotement in the improvement of MA’s core material (the remnant magnetization, the charge density of electret, and the magnetoelectric coupling coefficient) and the magnetic sensor with better performance can be helpful for the MA transmitting system.

**Table 3:** Results of signal sending and receiving experiments

Scheme Type	Core Material	Size	Frequency Range	Magnetic Flux Density	Efficiency (η)/Q factor (Q)	Reference
Electret-based MA	FEP	5 cm	0-200 Hz			(Cui et al., 2019a)
	FEP/THV	6 cm	20Hz	100 pT (0.4 m)		(Cui et al., 2022)
Magnet-based MA	N55 NdFeB	13 cm	1031 Hz		Q: 62.2	(Prasad et al., 2019a)
	N42 NdFeB	1.9 cm	30 Hz	10 μT (0.3 m)		(Strachen et al., 2018a)
	NdFeB Steel		0–1.6 kHz	50 pT (5 m)		(Golkowski et al., 2018)
	NdFeB	135 cm		47 μT (50 m)		(Fawole and Tabib-Azar, 2017)
	NdFeB	15 cm	30 Hz	30 nT (10 m)		(Liu et al., 2021)
	N42 NdFeB	1.6 cm	100-500 Hz	800 fT (100 m)		(Burch et al., 2018)
	NdFeB Array	3 × 5 × 7 cm	713Hz	0.7 fT(1 km)	Q: 70	(Fereidoony et al., 2022)
	NdFeB Resonator	1.27 × 5.08 cm	166Hz	1.09 fT(1 km)		(Thanalakshme et al., 2021)
Piezoelectric-based M	LiNbO_3_	9.4 cm	35.5 kHz		η: 1 × 10^−8^ to 2 × 10^−7^ Q: 3 × 10^5^ to 6 × 10^5^	(Kemp et al., 2019)
	PZT	8 cm	33.2 kHz	40 fT (6 m)		(Hassanien et al., 2019)
	PZT	6 cm	25/35 kHz	100 pT (5 m)		(Cao et al., 2022)
	PZT Metglas	10 cm	30 kHz	1 nT (0.9 m)	η:6.6 × 10^−16^	(Xu et al., 2019a)
	PZT Metglas	10 cm	23.95 kHz	0.1 pT (120 m)		(Dong et al., 2020)
	PZT Metglas Array	6 cm	22.4/24.2/26.3 kHz	1 nT (1.6 m)		(Zhang et al., 2022)
	PZT Terfenol-D	3.8 cm	37.95 kHz	1 nT (30 m)	η: 1.37 × 10^−17^ Q: 13.3	(Niu and Ren, 2022)
	PZT Metglas Ni Coil	15 cm	29.46/30.08 kHz	1.2 pT (170 m)	η:3.48 × 10^−12^	(Xiao et al., 2022)

In terms of radiation intensity, relying on the high remanence and other magnetic properties of NbFeB, magnet-based MA have inherent advantages in magnetic flux density in the near field, as compared with other types of MA. This makes it possible to use a smaller core unit to generate higher intensity magnetic field radiation.

However, due to the difference in the radiation model of the electret- and magnet-based MA, the attenuation rate of electret-based MA is relatively slow in the near field. Therefore, if the radiation intensity of an electret-based MA can be increased, the electret-based MA will have more advantages in effective propagation distance. If it is difficult to increase the charge density of a single-layer electret, the radiation intensity can be improved by improving the electret material structure, improving the electret polarization method, and using stacking technology (Chen et al., 2015; Rychkov et al., 2011). Moreover, the radiation intensity of the electret-based MA is proportional to its radiation frequency, so at a higher frequency, the radiation intensity of the electret-based MA will be further improved. At present, the performance of the third-generation rare earth permanent magnet materials such as NbFeB has approached its theoretical limit, and the development of permanent magnet performance has fallen into a bottleneck, so it is difficult to achieve substantial improvement (Bai et al., 2007; Brown et al., 2002; Cui et al., 2018b; Hu, 2014; Sprecher et al., 2014).

In terms of frequency and bandwidth, the three types have their own advantages. Electret-based MA and magnet-based MA are limited by the performance of driving mechanisms such as motors and are suitable for working at SLF range. Although there have been some studies on frequency-doubling technology, a certain amount of radiation intensity needs to be sacrificed. For piezoelectric-based MA, in order to achieve the maximum amplitude, piezoelectric materials all work near their natural frequencies, while the natural frequencies of conventional piezoelectric materials used in current research are all on the order of tens of kilohertz. Therefore, the radiation signal of the piezoelectric-based MA is in the VLF range. Among electret-, magnet-, and piezoelectric-based MA, since the radiation intensity of magnet-based MA is independent of frequency, in frequency modulation, the magnet-based MA has the widest absolute bandwidth, and the electret-based MA is the second. Because the piezoelectric-based MA is limited by the resonance characteristics of the material, its absolute bandwidth is the narrowest.

In terms of working stability, the existing magnet-based MA mostly uses sintered NdFeB as a core unit, and its working temperature generally does not exceed 80°C (Ma et al., 1994; Sepehri-Amin et al., 2012; Woodcock et al., 2012). If higher remanence is required and high-grade sintered NdFeB is used, the working temperature will be further reduced. The working temperature of common electret materials such as PTFE and FEP can reach hundreds of degrees Celsius (Hirschberg et al., 2018). Therefore, the electret-based MA is more suitable for harsh and complex working environments.

In addition, in terms of application prospects, the flexibility and bendable characteristics of the electret material are beneficial to the structure and shape design of the antenna (Xie et al., 2020; Zhu et al., 2018). Different electret antennas can be designed according to different antenna performance indicators, such as directivity and gain. This makes it possible to develop flexible conformal antennas based on electret antennas, enabling electret antennas to be implanted in wearable devices, aircraft, etc. and to play an important role in individual combat, aerospace, and other fields.

### Modulation schemes of mechanical antennas

The principle of radiation generated by mechanical motion makes the MA naturally fit the direct modulation method. At present, in research into MA, widely used signal modulation schemes include frequency-shift keying (FSK), minimum shift keying (MSK), amplitude shift keying (ASK), and polarization modulation. The signal modulation is achieved by directly changing the radiation of the antenna.

#### FSK

For rotating-electret-based MA and rotating-magnet-based MA, the radiation frequency corresponds to the rotation frequency. By changing the rotation frequency of the motor, the switching of the data signal between “0” and “1” is realized by switching between the “high” and “low” speeds of the motor. This is the 2FSK modulation method. Furthermore, multiple FSK modulation can be realized by expressing multi-bit data with multiple different frequencies (Glickstein et al., 2020; Wei and Bin, 2020). At present, the frequency modulation scheme is the most common approach for rotating MA. However, this scheme requires frequent changes of the motor speed, which severely tests the performance of the motor, and it is difficult to achieve signal modulation with a higher communication rate.

**Figure 9:**
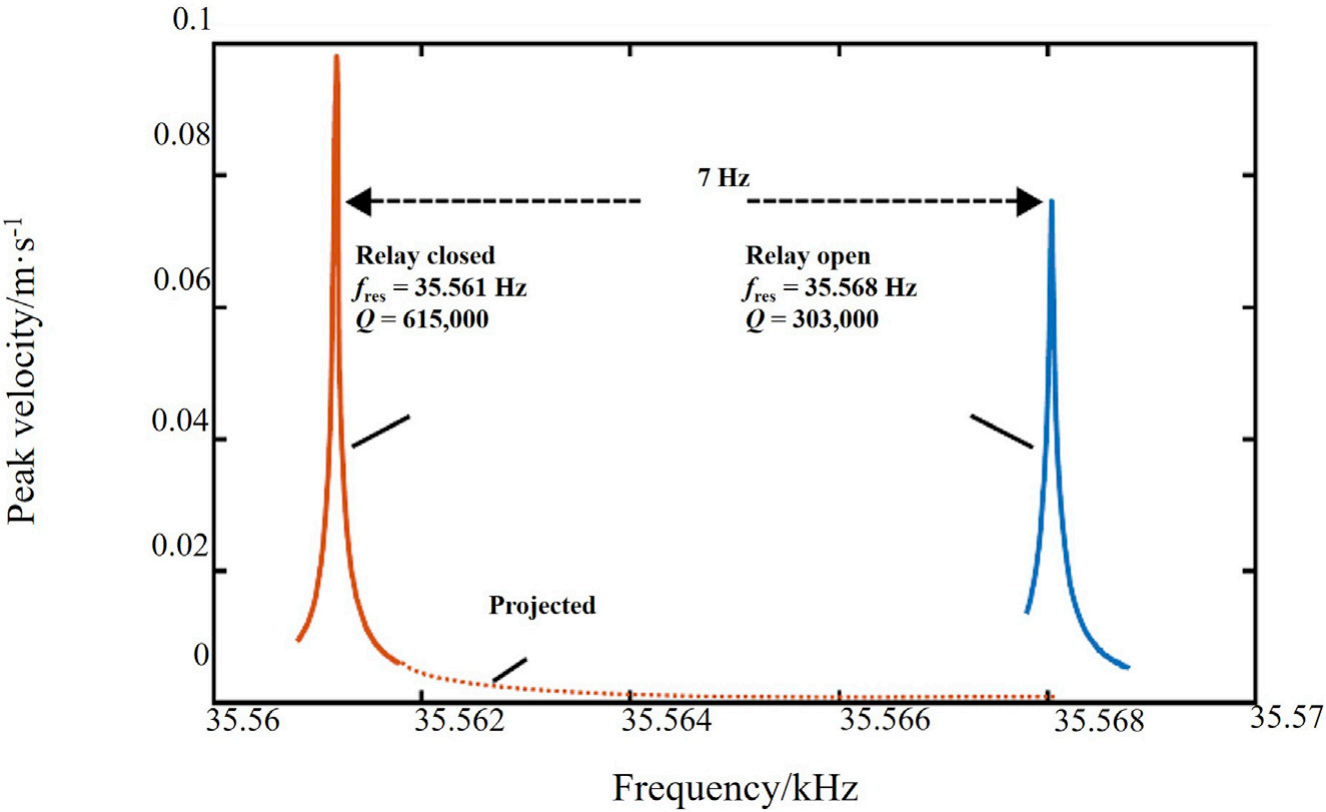
Schematic diagram of frequency modulation of piezoelectric MA designed by Mark A. Kemp et al Figures reproduced from: (Kemp et al., 2019) Copyright 2019, Nature Publishing Group.

Unlike rotating MA that relies on rotating mechanisms, piezoelectric-based MA is driven by electrical signals. The modulation of electrical signals is less restrictive. Mark A. Kemp et al. gave the piezoelectric crystal two resonance peaks by changing the presence or absence of external capacitors, and the corresponding frequencies of the two peaks are the “0” and “1” of the signal, as shown in Figure 9. Then the drive system changes the frequency of the excitation signal so that the antenna radiation signal can be converted between the “0” and “1”. However, the change in the resonance peak frequency is accompanied by a change in the amplitude. To ensure smooth signal reception, the amplitude of the two signals must be kept within a narrow range, which limits the frequency gap between the two signals, so the absolute bandwidth is narrow.

Ahmed E. Hassanien et al. took two points with the same amplitude on both sides of the peak of the piezoelectric material frequency response curve as the corresponding points of the "0" and "1" signals. It is easier to achieve 2FSK modulation under the premise of acceptable radiation intensity. How well the frequency modulation scheme of the piezoelectric MA performs is also limited by the quality of the piezoelectric material (Strachen et al., 2018a). When the modulation speed is too high, the high-speed switching of the piezoelectric material between different resonance frequencies will reach its limit, which will unbalance the edge speed of the resonator and the distort signal after demodulation.

#### MSK

**Figure 10:**
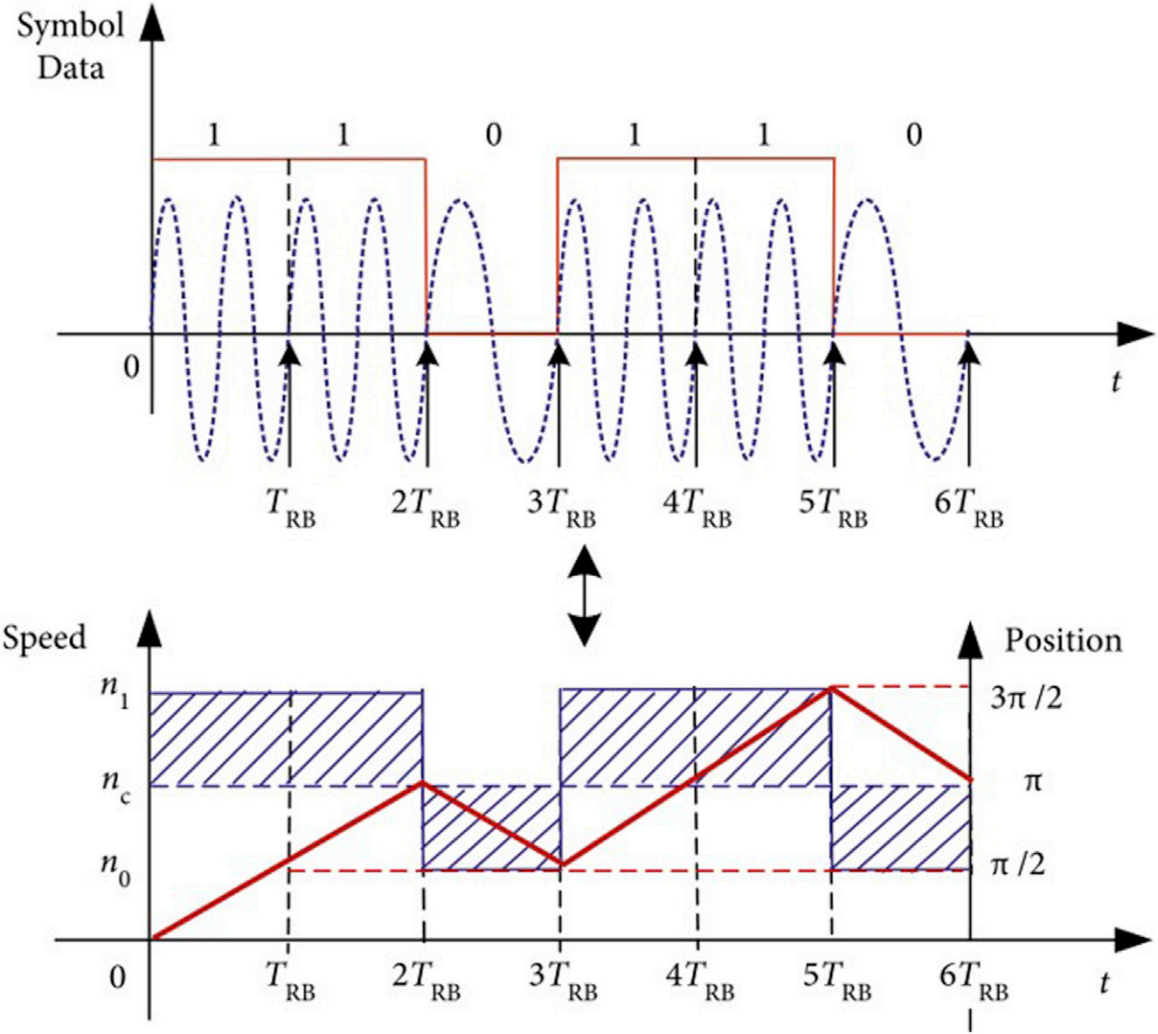
Schematic diagram of MSK for MA designed by Xie He et al. Figures reproduced from: (He et al., 2022) Copyright 2022, Multidisciplinary Digital Publishing Institute (MDPI).

As for the MA driven by motor, the rotational speed cannot be shifted abruptly due to the limit of the motor performance and the rotational inertia, but the modulated waveform always needs to have constant amplitude and continuous phase. As a result, MSK can be applied for the modulation of MA to reduce distortion. Qiang Zhou et al. proposed the technology scheme and information-loading method of MSK for the rotating-magnet-based MA (Wei and Bin, 2020). Xie He et al. investigated the performance analysis of MSK on MA in Figure 10 for the first time and proposed the control strategies of the motor initially including characterizing the effects of the step response generated by the motor on the information loading. Furthermore, the results show that the transmission capacity can be enhanced by increasing output torque or decreasing inertia load (He et al., 2022). This scheme can be effective in the system design and practical implementation of MA with a suitable control strategy.

#### ASK

**Figure 11:**
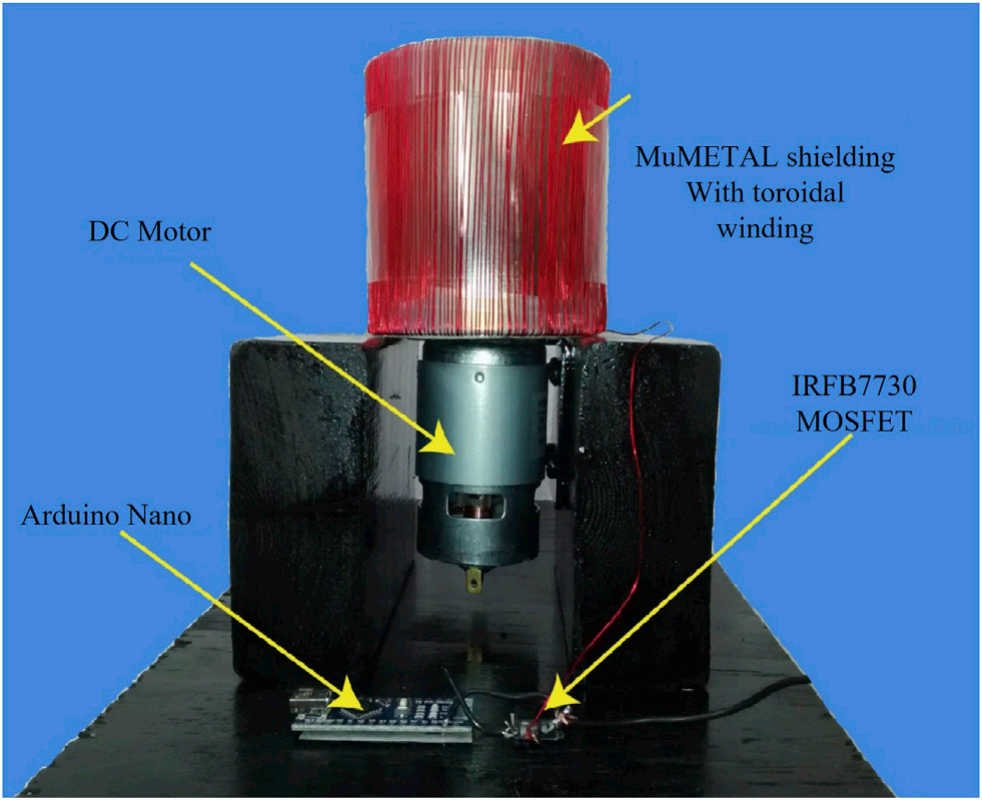
Amplitude modulation scheme designed by Nathan Strachen et al. Figures reproduced from: (Strachen et al., 2018a) Copyright 2018, Public Library of Science(PLOS).

Amplitude modulation is another widely used modulation scheme in the field of MA. By artificially controlling the magnetic environment near the antenna, the external radiation amplitude of the antenna can be changed regularly. In research on rotating-magnet-based MA, Nathan Strachen et al. from the University of Wisconsin-Madison changed the magnetic resistance of the shielding mechanism near the magnets by applying magnetic shielding and using the shielding coil current to modulate the magnetic field amplitude, as shown in Figure 11 (Strachen et al., 2018b). This scheme does not need to change the rotation frequency, avoids the dependence of the radiation frequency on the performance of the motor, and achieves a higher frequency signal modulation for the rotating-magnet-based MA.

#### Polarization modulation

Duojia Zhang from Xi’an University of Technology chose polarization modulation as the modulation scheme for MA (Zhang, 2019; Zhang et al., 2020). The realization principle of this scheme is similar to amplitude modulation. By adding a modulator that rotates coaxially and asynchronously with the permanent magnet, the magnetic resistance of the environment around the antenna is changed so that the magnetic field in the direction corresponding to the antenna and the modulator is greatly attenuated, while in the direction perpendicular to the modulator, the magnetic field is basically unchanged. The magnetic field in the direction corresponding to the modulator has high attenuation, and the magnetic field in the direction perpendicular to the modulator is basically unchanged. The magnet maintains a fixed rotating frequency. As the modulator changes its rotating position according to the data signal, the receiving end receives magnetic signals with different polarization directions. This scheme also avoids the high-frequency switching of the motor speed, but the addition of a modulator makes its mechanical structure more complicated and the system bulkier.

## Conclusions and future research directions

Aiming at the bottleneck of excessive size and power consumption of traditional LF transmitters, MA is proposed to break the limit that the size of the LF transmitting antennas need to be in the same order of magnitude with the wavelength of the transmitted signal. Consequently, LF transmitters based on MA technology have gradually become the hotspot of this research field, on which plenty of attention and effort have been applied by the universities and research institutions around the world. This work presents a comprehensive and critical survey of SLF transmitters based on MA. The results of theory and experiments in related work have demonstrated the correctness and feasibility of LF transmitter with the MA concept.

However, at the current research level, although plenty of efforts have been applied on MA around the world, there still exist many challenges in this field:

(1) Challenge on theory analysis. At present, MA is usually equivalent to an electric or magnetic dipole for theory analysis. This simplification can be convenient for calculation; however, the internal conditions of the core material is not taken into consideration, such as charge distribution, polarization effects, magnetoelectric coupling, etc., which cannot reveal the correlation law between the micro characteristics of the material and the macro performance of the MA.
(2) Challenge on material preparation. The core material plays the crucial roles in the performance of MA. As for the electret-based MA, electret with the high charge density is indispensable to obtain a measurable result. However, the conditions for electret to carry high charge are harsh; besides careful electret selection, it is also necessary to comprehensively adjust various and complex polarization conditions such as temperature and voltage. What’s more, for magnetoelectric MA, the manufacturing process for magnetostrictive layer with a single domain and piezoelectric layer with a strong driving force is complicated and difficult, which is a challenge to improve the radiation efficiency.
(3) Challenge on antennas performance. The radiation intensity of MA is limited due to factors such as antenna volume, power consumption, and driving devices, though several improved implementation schemes and research progress on the MA have been proposed by different research group and achieved specific certain results. In addition, the problems of miniaturization and the high sensitivity of extremely weak magnetic field reception technology have not been solved either, and the application scenarios of MA are restricted seriously because of the work distance within a range of 1 km or less.

As for the future direction, the expectation of MA is summarized as follows.

(1) In the future research, for long-distance LF communication scenarios, how to increase the radiation intensity at the expense of a certain amount of compactness and portability will become the focus of research.
(2) In addition, in view of the low communication rate of existing SLF communication systems, which information loading and signal modulation methods to adopt will also be a problem worthy of attention.
(3) Furthermore, on account of the strong penetration in lossy media of LF band, besides communication, the LF functions based on MA technology such as the detection and positioning underwater, underground, and indoor can be developed in the next step, which were previously limited by the size and power consumption of the LF transmitters.
